# Updating a Strategy for Histone Deacetylases and Its Inhibitors in the Potential Treatment of Cerebral Ischemic Stroke

**DOI:** 10.1155/2020/8820803

**Published:** 2020-09-05

**Authors:** Yuzhen Xu, Qian Wang, Jianxin Chen, Yihong Ma, Xueyuan Liu

**Affiliations:** ^1^Department of Neurology, Shanghai Tenth People's Hospital, Tongji University School of Medicine, No. 301 Middle Yanchang Road, Shanghai, China; ^2^Department of Central Laboratory, Taian City Central Hospital, Shandong First Medical University & Shandong Academy of Medical Sciences, Taian, Shandong Province, China; ^3^Department of Neurology, Jinan First People's Hospital, Shandong Traditional Chinese Medicine University, Jinan, Shandong Province, China; ^4^Department of Neurology, Graduate School of Medical Sciences, Kumamoto University, Kumamoto, Japan

## Abstract

**Background:**

Cerebral ischemic stroke is one of the severe diseases with a pathological condition that leads to nerve cell dysfunction with seldom available therapy options. Currently, there are few proven effective treatments available for improving cerebral ischemic stroke outcome. However, recently, there is increasing evidence that inhibition of histone deacetylase (HDAC) activity exerts a strong protective effect in in vivo and vitro models of ischemic stroke. *Review Summary*. HDAC is a posttranslational modification that is negatively regulated by histone acetyltransferase (HATS) and histone deacetylase. Based on function and DNA sequence similarity, histone deacetylases (HDACs) are organized into four different subclasses (I-IV). Modifications of histones play a crucial role in cerebral ischemic affair development after translation by modulating disrupted acetylation homeostasis. HDAC inhibitors (HDACi) mainly exert neuroprotective effects by enhancing histone and nonhistone acetylation levels and enhancing gene expression and protein modification functions. This article reviews HDAC and its inhibitors, hoping to find meaningful therapeutic targets.

**Conclusions:**

HDAC may be a new biological target for cerebral ischemic stroke. Future drug development targeting HDAC may make it a potentially effective anticerebral ischemic stroke drug.

## 1. Instruction

Cerebral ischemic stroke is a serious neurological disease that leads to morbidity, mortality, and severe long-term disability [1]. Because of its high basal metabolic rate, dependence on maintained ion gradients, and high concentrations of glutamate and aspartic acid, central nervous system neurons are particularly vulnerable to the direct effects of hypoglycemia and hypoxia [2–4]. The subsequent Na^2+^ influx and Ca^2+^ overload result in cytotoxic edema, acidosis, organelle, and necrotic cell death finally [5]. The endogenous neuronal repair occurred in the site of the penumbra, including neurogenesis and neovascularization, which appear days to weeks after an ischemic stroke [6]. It determines the final degree of tissue regeneration and function recovery [7]. Ischemic stroke has a complex pathophysiological process involving excitotoxicity, oxidative stress, and inflammation [8]. Therefore, effective treatments should have a multitarget effect to achieve the purpose of reducing nerve damage and promoting tissue repair [9, 10].

Histone deacetylase (HDAC) is a significant enzyme that regulates intracellular protein acetylation. Abnormal HDAC activity is associated with many diseases. HDAC is an essential drug target, many of which have been developed into clinical drugs. Recently, preclinical studies have confirmed that HDAC and its inhibitor (HDACi) are potential drugs for the treatment of stroke [11]. HDAC and HDACi regulate a series of processes from cell survival to regeneration, significantly reduce infarct area, and play a neuroprotective role in vivo and vitro [10]. Here, our review will discuss the role of histone deacetylases (HDACs) in cerebral ischemic stroke and update the potential therapeutic strategy of HDACi as emerging drugs in stroke treatment in recent times.

## 2. Overview of the HDACs and HDACi in Cerebral Ischemia

### 2.1. Overview of the HDACs

**Figure 1:**
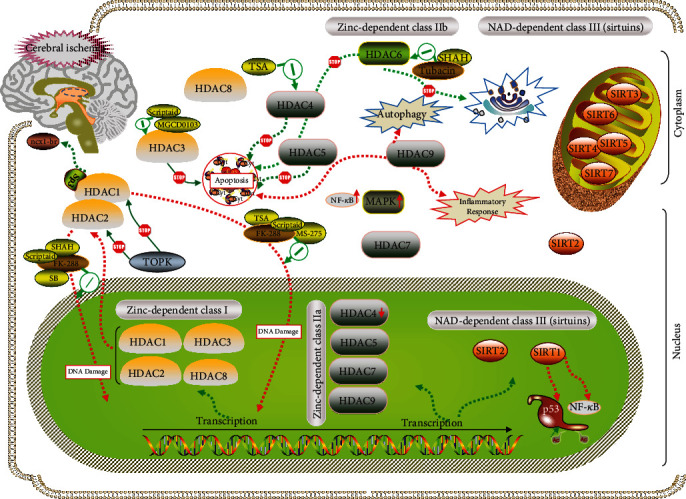
Overview of the probable mechanism between HDACs and their inhibitors. Due to the different distributions of HDACs in the cytoplasm and nuclei, the related mechanisms of cerebral ischemia show the different types. When cerebral infraction occurred, HDAC 1 and 2 in the nucleus were released into the cytoplasm that caused homeostasis of the cellular environment to be destroyed. It will continuously lead to the degradation of DNA. Meanwhile, HDAC 3, 4, 5, and 9 would participate in apoptosis, inflammation response, and Golgi apparatus dysfunction as well as autophagy, which is particularly apparent in HDAC9, respectively. Moreover, HDAC NAD^+^-dependent class III (SIRT1) could aggravate the ischemic injury p53 acetylation and NF-*κ*B.

HDACs are conservative evolutionary enzymes involved in the epigenetic regulation of gene expression and protein functions [12]. To date, 18 potential HDACs have been identified in mammalian cells, and all of them are expressed in the adult brain [13]. Given that histone modification can regulate chromatin structure and gene expression in which abnormal alteration appearance in HDACs is associated with cerebral ischemic development, the mechanism of action between HDACs and cerebral infarction is summarized in Figure 1. Based on function and DNA sequence similarity, HDACs are organized into four different subclasses (I-IV) [14]: the zinc-dependent class I (HDAC 1, HDAC 2, HDAC 3, and HDAC 8), the zinc-dependent class II (class IIa: HDAC 4, HDAC 5, HDAC 6, HDAC 7, and HDAC 9; class IIb (HDAC6), the NAD-dependent class III (sirtuins), and class IV (HDAC 10 and HDAC 11). The class I HDACs display an extensive presence in nuclei. In addition to nuclear localization, the expression of class I isoforms has also been observed in cytoplasmic domains. The class I HDACs are a large class of constitutive nuclear proteins, while class II HDACs, divided into classes IIa and IIb, mainly shuttle between the nucleus and cytoplasm. Class IV HDACs are generally composed of a member, HDAC11, and little is known about its role in ischemic stroke. Sirtuins have been discussed in a recent review, referred to as class III HDACs, which are structurally and enzymatically distinct NAD-dependent enzymes [15–18].

### 2.2. Overview of the HDACs

According to the HDAC classification, currently, the most common HDACi used in cerebral ischemic models are class I and class II inhibitors. Here, we summarized the related HDACi in cerebral ischemia. Class I is including MS-275 [19], Scriptaid [8],, trichostatin A (TSA) [20], suberoylanilide hydroxamic acid (SAHA) [21, 22], valproic acid (VPA), sodium butyrate (SB) [23], and 4-phenylbutyric acid (4-PB) [24]. Besides that, nicotinamide is a nonspecific inhibitor of class III HDACs [25]. Here, we summarized the publication of HDAC and HDACi in recent years as a treatment strategy and probably mechanism in cerebral ischemia (Table 1).

## 3. HDACs in Cerebral Ischemia

### 3.1. Zinc-Dependent Class I

HDAC1 is the first mammalian protein identified to have histologically oriented deacetylase activity [26], which exerts an essential role in regulating the cell cycle and is necessary for the transcriptional suppression of cell cycle genes. The association between HDAC1 and the promoter region of specific genes is related to their transcriptional suppression. The functional acquisition of HDAC1 provides adequate protection against DNA damage and neurotoxicity of cultured neurons, which is used as a model of ischemia in vivo [27]. The penumbra of HDAC2 in the rat cerebral cortex enhanced 4 or 24 hours after photothrombotic stroke (PTS). PTS increases the expression of HDAC1 and HDAC2 in the penumbra, which leads to the redistribution of HDAC1. However, the redistribution of HDAC2 from the nucleus to the cytoplasm is less than that of HDAC1 [28]. In transient middle cerebral artery occlusion (tMCAO) models of rats, the specificity protein family of transcription factor 3 (Sp3) together with HDAC1/HDAC2 complex could modulate the acetylation of ncx1 brain promoter (ncx1-br), which may act as an innovative development strategy in stroke treatment intervention [29].

Furthermore, T-LAK-cell-originated protein kinase (TOPK) has a neuroprotective effect on cerebral ischemia-reperfusion injury by inhibiting HDAC1/HDAC2 activity, which may be related to the neuroprotective effect of TOPK on cerebral ischemia-reperfusion injury [30]. The expression of HDAC3 in the pathological process of diabetes and stroke can protect the brain from ischemia-reperfusion (I/R) injury by regulating oxidative stress, apoptosis, and autophagy in vivo and in vitro, and the mechanisms may be by the upregulated expressions of brain and muscle ARNT-Like 1 and AIM2 inflammatory bodies [31, 32]. Calpeptin pretreatment blocked the attenuation of the nuclear distribution of HDAC3 in vivo [33]. HDAC8 plays a protective role in the cytotoxicity of renal cell death [34], but its effect on cerebral ischemia was not obvious.

### 3.2. Class II HDACs

In ischemic stroke models, HDAC4 expression is reduced, while HDAC4 may reduce neuronal apoptosis by decreasing high-mobility group box 1 (hMGB1) protein expression and promote angiogenesis and nerve regeneration through the release of VEGF signal of hypoxia-inducible factor-1 [35–37]. HDAC4's interactive partners such as MEF2, ATF4, and NF-*κ*B may also mediate the roles of attenuation of neuronal apoptosis and promotion of angiogenesis and neurogenesis of ischemic stroke [38]. A recent study found that HDAC5 effectively inhibits the involvement of myocardial-associated transcription factor A (MRTF-A) in IR-induced apoptosis of cortical neurons [39]. In one study, miR-217 promotes the accumulation of HDAC5 in the nucleus and affects the cerebral ischemia-reperfusion injury via the miR-217/MEF2D/HDAC5 axis [40].

HDAC9 is expressed in the cerebral and systemic arteries. In rat cerebral ischemia/reperfusion injury, the brain injury can be alleviated by silencing the HDAC9 gene by lentivirus recombination in the brain with upregulated HDAC9. It is involved in hypoxia-glucose-induced dysfunction of cerebral microvascular endothelial cells, increased inflammatory response, apoptosis, endothelial cell permeability disorder, and decreased expression of tight junction protein [41]. HDAC9 deficiency and downregulation of phosphorylated IkBa are related to the role of phosphorylated nuclear factor-kappa B (NF-*κ*B) and mitogen-activated protein kinase (MAPKs), including regulating phosphorylated p38, phosphorylated extracellular signal-regulated kinase 1, ERK1/2, and phosphorylated c-Jun n-terminal kinase, in promoting ischemic brain injury [42]. In addition, HDAC9 polymorphism can be used as a biomarker of susceptibility, severity, and short-term prognosis of atherosclerotic stroke [43]. However, Su et al. [44] indicate that variant rs2107595 of HDAC9 may have no association with higher ischemic stroke risks in southern Han Chinese.

HDAC6 can inhibit the survival and regeneration of neurons [18]. On the other hand, HDAC6 inhibits ODG reperfusion-induced tubulin deacetylation and caspase-3 activation and protects Golgi apparatus breakage and apoptosis induced by oxyglucose deprivation reperfusion (OGDR) [45]. Another research exhibits tripartite inhibition of HDAC6 that showed neuroprotection against various cellular insults [46]. Pharmacological or gene inhibition experiments on HDAC6 confirmed the block caused by stroke, indicating that the normal function of HDAC6 is necessary for the efficacy of rehabilitation treatment after stroke [47].

### 3.3. NAD-Dependent Class III (Sirtuins)

Sirtuins are a group of histone deacetylase whose activity is dependent on and regulated by nicotinamide adenine dinucleotide (NAD^+^) [48]. In ischemic brain injury, the latest research shows that the decrease of SIRT1 activity may aggravate ischemic brain injury in permanent cerebral ischemia mice, whether by pharmacological or genetic methods [49]. This neuroprotective effect may be mediated by SIRT1 inhibition or deletion through p53 acetylation [50] and NF-*κ*B. Besides, the use of the SIRT1 activator can also reduce ischemic brain injury. SIRT3 has been reported to protect neurons from n-methyl-d-aspartate (NMDA) excitotoxicity, suggesting that SIRT3 may also play a beneficial role in cerebral ischemia. However, it is necessary further to illuminate the role of SIRT3 in cerebral ischemic injury [51].

### 3.4. HDACi in Cerebral Ischemia

**Figure 2:**
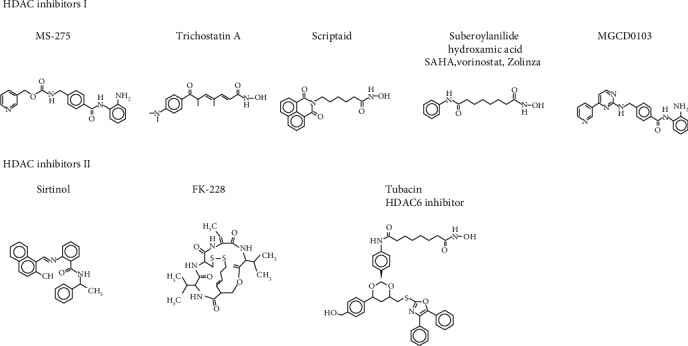
Primary structural formula of HDAC inhibitors in cerebral ischemia.

In order to explore the usefulness of HDAC inhibitors, we discussed and summarized the mechanism and show the structural formula of the dominant HDAC inhibitors in vitro and in vivo, which is closely related to cerebral ischemia for brain protection (Figure 2).

### 3.5. HDACi Class I

MS-275 (entinostat), a class I histone deacetylase inhibitor (HDAC), modulates differentiation and prevents brain injury [52]. In stroke model MS-275, a neuroprotective effect had been demonstrated in vitro [53]. In ischemic pathology, p53 plays a role, which is regulated by class I HDA in processes of acetylation [54]. Sean P et al. had found that MS-275 is not responsible for modulating the activity of p53. Unlike affecting p53, the evidence showed the effect of mitochondria for protection in the cerebral ischemia model. TSA has been shown to have neuroprotective properties in neurons [55] through blocking p53 accumulation after DNA damage. Unlike MS-275, it had shown different mechanisms and manifestations in the role of brain protection. SHAH is an FDA-approved HDACi. Its has an anti-inflammatory effect and is carefully used for protection by early treatment of the cerebral ischemia mouse model, closely related to its effect of reducing microglial activation and priming these activations toward an M2 phenotype [22].

### 3.6. HDACi Class II

Compared with traditional HDAC inhibitor, Sirtinol, as a SIRT1-specific inhibitor and activator [56], significantly reversed the effect of resveratrol postconditioning on cerebral ischemia in a mouse model [57]. Otherwise, FK228 a kind of selective HDAC1/2 inhibitor could help restrain TOPK through the AKT pathway to attenuate the oxidative stress in the early stage of ischemic stroke. Besides that, in 2019, tubacin selectively inhibits HDAC6 activity which had been identified in transient occlusion of the middle cerebral artery by ameliorating endothelial dysfunction [58].

## 4. Summary and Perspective

Studies over the past few decades have shown that HDACs play an essential role in the development of cerebral ischemic stroke by reversibly regulating protein acetylation. As a histone acetylation remover and a key regulator of epigenetics, HDACs have been found to have abnormalities and dysfunctions in ischemic stroke, which provides a novel and attractive target to intervene. Also, HDAC has some anti-inflammatory effects and is involved in the mechanism of brain protection. As a target of an early inflammatory reaction, it acts on HDACi and plays a neuroprotective role. One of the inhibitors had been showing the protection against ischemic brain injury in the cerebral ischemia mouse model through increasing protein acetylation levels in the mouse brain. However, in human plasma, at the early stage of other diseases, such as chronic hepatitis B, HDAC activity abnormalities were found in patients with other diseases, and their levels were related to the severity of the disease [59]. However, the exact function of HDACs as a central medium for proliferation and regulation remains a mystery. At present, the biomarker of fresh blood has been applied and can be used as a clear indicator for cerebral ischemic prediction [60]. However, at present, no detailed research on HDAC is related to plasma, and CSF biomarkers had been explored in cerebral infarction.

Intriguingly, valproic acid, which is commonly prescribed as an anticonvulsant drug, was subsequently discovered to be an HDAC inhibitor [61]. In cellular and animal models, VPA has been shown to have neuroprotective properties at the therapeutic level. Valproic acid (VPA), a widely used antiepileptic mood stabilizer drug, has been shown to have neuroprotective effects against various injuries through a variety of signal pathways, such as blocking the increase in caspase-3 activity. VPA significantly upregulated the expression activity of ERK and Akt [62]. Recently, new research also found that VPA could inhibit the enzyme GSK3 for neuroprotective properties, which are related to the drug anti-inflammatory activity [63]. Like VPA, SB inhibits cell growth through cell cycle arrest, while stable transcriptional inhibition of cell cycle promotes the reduction of microglia and inhibition of other inflammatory markers in ischemic brain [55, 64]. 4-Phenylbutyrate (PBA) has the effect of anticerebral ischemia by inhibiting endoplasmic reticulum stress-mediated apoptosis and inflammation [65]. The effect of these inhibitors may be related to the inhibition of HDAC, which leads to high acetylation of chromatin proteins and changes in gene expression. Besides that, TMP269 is a particular class IIA histone deacetylase inhibitor that has a protective role by upregulating the level of histone 2 acetylation in tMCAO mouse models, in the meantime, which upregulates the expression of tissue kallikrein [66]. Recently, one study has also indicated that SIRT2 inhibitor AGK2 can alleviate reperfusion injury of a myocardial ischemia model [67]. Because there are many common pathological mechanisms between myocardial and cerebral ischemia-reperfusion injuries, the inhibition of SIRT2 may also have a neuroprotective effect in cerebral ischemia [68]. Furthermore, tubastatin A (TubA) is a new type of HDAC6 inhibitor, which can improve the functional outcome, reduce cerebral infarction, and attenuate neuronal apoptosis in MCAO rats by downregulating fibroblast growth factor-21 (FGF-21) [69].

M344 is a kind of HDAC inhibitor, which led to a remarkable decrease in the phosphorylation of JNK and c-Jun, concomitant with a significant abrogation of apoptosis caused by potassium deprivation in cultured cerebellar granule neurons [70], but within the current scope of treatment for cerebral infarction, the function between HDAC and HDACi can play a minimal role. Although many protective effects have been achieved in animals, there are still many defects in clinical trials for therapy using them. In recent years, the use of HDACi alone is improbable to have a substantial impact in the clinic, and these promising drugs may lie in cognitive combination therapy in the future. A large number of reasonable, HDAC-based combinations are possible with the gradual elucidation of the multiple action mechanisms of these drugs and the emergence of new combinations. In the field of treatment, HDAC-based combination therapy has made the fastest progress, including cytotoxic chemotherapy. More studies are needed to systematically analyze the role of a single HDAC in different types and stages of ischemic stroke. Further elucidation of the mechanism of action of HDACs and HDACi will provide a bright prospect for HDACi as one of the many tools to combat stroke.

## Figures and Tables

**Table 1:** Primary HDAC and HDAC inhibitors in cerebral ischemia.

HDAC classification		HDAC inhibitors	Location
Zinc-dependent class I	HDAC 1	MS-275, FK-288, TSA, VPA, Scriptaid	NU
	HDAC 2	FK-288, SAHA, SB, Scriptaid	NU
	HDAC 3	MGCD0103, Scriptaid	NU/CM
	HDAC 8	/	NU/CM

Zinc-dependent class II (class IIa)	HDAC 4	TSA, 4-PBA	NU/CM
	HDAC 5	/	NU/CM
	HDAC 7	/	NU/CM
	HDAC 9	/	NU/CM

Zinc-dependent class II (class IIb)	HDAC 6	Tubacin, SAHA	CM

NAD^+^-dependent class III (sirtuins)	SIRT1	Sirtinol	NU
	SIRT2	AKG2	NU/CM
	SIRT3	/	Mitochondria

NU: nucleus; CM: cytoplasm.

## Data Availability

The data used to support the findings of this study are available from the corresponding author upon reasonable request.
